# Reflectance Imaging Spectroscopy (RIS) for *Operation Night Watch*: Challenges and Achievements of Imaging Rembrandt’s Masterpiece in the Glass Chamber at the Rijksmuseum

**DOI:** 10.3390/s21206855

**Published:** 2021-10-15

**Authors:** Francesca Gabrieli, John K. Delaney, Robert G. Erdmann, Victor Gonzalez, Annelies van Loon, Patrick Smulders, Roy Berkeveld, Robert van Langh, Katrien Keune

**Affiliations:** 1 Conservation and Science Department, Rijksmuseum, Hobbemastraat 22, 1017 ZC Amsterdam, The Netherlands; R.Erdmann@rijksmuseum.nl (R.G.E.); gonzalvic@gmail.com (V.G.); A.van.Loon@rijksmuseum.nl (A.v.L.); R.van.Langh@rijksmuseum.nl (R.v.L.); k.keune@rijksmuseum.nl (K.K.); 2 Scientific Research Department, National Gallery of Art, 6th and Constitution Avenue NW, Washington, DC 20565, USA; J-Delaney@nga.gov; 3 Conservation and Restoration, University of Amsterdam, Johannes Vermeerplein 1, 1071 DV Amsterdam, The Netherlands; 4 Paintings Conservation Department, Mauritshuis, Plein 29, 2511 CS The Hague, The Netherlands; 5 TBRM Engineering Solutions (Former Segula Technologies), De Witbogt 2, 5652 AG Eindhoven, The Netherlands; p.smulders@tbrm-es.nl (P.S.); roy@cirolabs.com (R.B.); 6 Van’t Hoff Institute for Molecular Sciences, University of Amsterdam, Science Park 904, 1090 GD Amsterdam, The Netherlands

**Keywords:** reflectance imaging spectroscopy, hyperspectral sensors, cultural heritage, pigments identification, pigments mapping

## Abstract

Visible and infrared reflectance imaging spectroscopy is one of the several non-invasive techniques used during *Operation Night Watch* for the study of Rembrandt’s iconic masterpiece *The Night Watch* (1642). The goals of this project include the identification and mapping of the artists’ materials, providing information about the painting technique used as well as documenting the painting’s current state and ultimately determining the possible conservation plan. The large size of the painting (3.78 m by 4.53 m) and the diversity of the technical investigations being performed make *Operation Night Watch* the largest research project ever undertaken at the Rijksmuseum. To construct a complete reflectance image cube at a high spatial resolution (168 µm^2^) and spectral resolution (2.54 to 6 nm), the painting was imaged with two high-sensitivity line scanning hyperspectral cameras (VNIR 400 to 1000 nm, 2.54 nm, and SWIR 900 to 2500 nm, 6 nm). Given the large size of the painting, a custom computer-controlled 3-D imaging frame was constructed to move each camera, along with lights, across the painting surface. A third axis, normal to the painting, was added along with a distance-sensing system which kept the cameras in focus during the scanning. A total of 200 hyperspectral image swaths were collected, mosaicked and registered to a high-resolution color image to sub-pixel accuracy using a novel registration algorithm. The preliminary analysis of the VNIR and SWIR reflectance images has identified many of the pigments used and their distribution across the painting. The SWIR, in particular, has provided an improved visualization of the preparatory sketches and changes in the painted composition. These data sets, when combined with the results from the other spectral imaging modalities and paint sample analyses, will provide the most complete understanding of the materials and painting techniques used by Rembrandt in *The Night Watch*.

## 1. Introduction

*The Night Watch* (1642) is Rembrandt’s largest (3.78 m by 4.53 m) and most famous painting. It was made for the arquebusiers’ guild hall in Amsterdam. The painting depicts a militia group of the seventeenth century preserving the city and public order in Amsterdam under the command of Captain Frans Banninck Cocq and his lieutenant Willem van Ruytenburgh, the men dressed respectively in black and yellow at the center of the composition. What makes the painting so unique is that it is the only militia portrait depicting the group in action. Rembrandt’s painting was one of the seven militia portraits hanging in the Kloveniersdoelen (arquebusiers’ headquarters), but in 1715 *The Night Watch* was transferred to Amsterdam’s town hall and it trimmed along the four sides to fit on the wall (between two doors) of the new location [1]. It has been hanging in the Rijksmuseum since 1885, except during World War II, when it was hidden.

*The Night Watch* has undergone several types of conservation treatments, such as wax-resin relining, varnish re-generation as well as the removal and re-vanishing of the painting [2]. Before 1975, when the latest and most documented conservation treatment was performed, *The Night Watch* had already been treated some 25 times. All of these conservation treatments have affected the surface of the painting [3]. In addition, *The Night Watch* has been subjected to several acts of vandalism. In 1911, and then again in 1975, the painting was attacked by a visitor with a knife. More recently, in 1991, the painting was attacked by a visitor with a spray bottle of acid. Due to the complex history of the painting (i.e., numerous treatments, local damages from attacks and normal degradation processes which have resulted in crusts, hazes and abrasions of the paint layers in places) as well as to the complex paint mixtures used by the artist, developing a detailed understanding of Rembrandt’s painting process has remained challenging.

**Figure 1:**
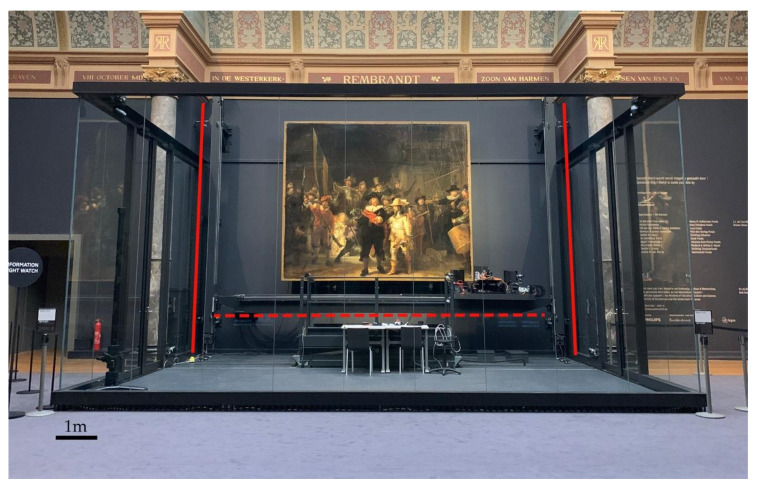
The Night Watch surrounded by the glass chamber in the Gallery of honor of the Rijksmuseum. The red vertical lines highlight the two y axes of the imaging frame, and a red-dot horizontal line highlights the x-beam to which the instrument platform is anchored.

In July 2019, the Rijksmuseum started a large conservation research project, *Operation Night Watch*, in order to study Rembrandt’s masterpiece in detail. The aims are to (i) characterize the materials used by Rembrandt and their distribution over the painting, (ii) document the condition of the painting by identifying ongoing degradation processes, with the goal of understanding their origin and preventing a future recurring, (iii) develop a science-based, well-founded conservation treatment plan. Given the large dimensions of *The Night Watch* and the importance of the painting to the visitors of the Rijksmuseum, a glass chamber that allows visitors to see the painting while the research is ongoing was built in the Gallery of Honor of the Rijksmuseum (Figure 1). *Operation Night Watch* involves a team of conservators, scientists, photographers, curators and engineers and is divided into two different phases. In the first phase, conservation and scientific investigations are being performed whose results will help to define a second phase, in which the conservation treatment of the painting, decided according to the scientific results and actual needs of the painting, will occur.

During the scientific research phase, many high-technology scientific instruments have been used to non-invasively analyze the painting at the macroscopic scale, such as macro X-ray fluorescence scanning (MAXRF), macro X-ray powder diffraction scanning (MAXRPD), high-resolution photography (visible and UV), optical coherence tomography (OCT), 3-D scanning and reflectance imaging spectroscopy (RIS). The results obtained with these techniques are being combined and integrated with the results from in-depth scientific studies at the micro-scale of paint samples.

This paper focuses on the RIS experimental set-up and examples of the results obtained from this spectral imaging modality. RIS, also known as hyperspectral imaging, was developed for the field of remote sensing and has now been adapted for the non-invasive study of cultural heritage objects [4]. RIS is known as a molecular technique because it gives information about molecules present in the paint layers. Specifically, information about electronic transitions occurs mainly in the visible spectral range, and information about vibrational modes (combinations and overtones) in the short-wavelength infrared. During the last decades, research has shown that when RIS results are combined with other complementary non-invasive imaging techniques [5,6,7], a more complete understanding of the composition polychrome artworks such as manuscripts, wall paints [8,9] and, in particular, paintings [10] can be obtained. Furthermore, RIS has been proven to be useful in the study of Rembrandt’s paintings to help in the identification and mapping of pigments as well as to visualize changes in the painted composition [11,12] in spite of their often dark appearance.

RIS is usually performed using push broom line scanning hyperspectral cameras, which consists of a relay lens and an imaging spectrometer that collects a spectrum for each spatial pixel along the slit of the spectrometer. When the hyperspectral camera is stationary, it provides an image of a vertical spatial line on the painting for each spectral band. To collect a 3-D image cube, the camera is translated across the surface of the painting, and the 3-D image cube (2-D spatial, 1-D spectral) is built up line by line [4]. The height of the spatial line on the painting and the number of spatial lines collected during the scan defines a swath, which corresponds to a single 3-D image cube acquisition. Note that there are advantages to moving the painting instead of the camera to build up the 3-D image cube, as it allows us to easily switch hyperspectral cameras and adjust the illuminating system. In the laboratory at the Rijksmuseum the cameras are kept stationary, and the painting is moved on a motorized easel. The focus can be checked and easily adjusted if necessary. A full RIS acquisition (400 to 2500 nm) consists of two separate data collections with two different hyperspectral cameras (VNIR from 400 to 1000 nm and SWIR from 900 to 2500 nm), along with the collection of the white and dark reference needed to convert the data to apparent reflectance. An estimate of how long it would take to scan the entire surface of *The Night Watch* can be made knowing that *The Night Watch* is about 18 m^2^ and that a full RIS acquisition of a 1 m^2^ painting at a spatial resolution of 200 µm takes about a day. Thus, it would take about 18 days or 1 month in working days for such a collection. Considering that the painting’s large size ruled out moving it relative to the hyperspectral cameras, it is not surprising that the RIS collection on *The Night Watch* presented many technical challenges.

Some of the challenges that had to be addressed during the scanning of such a large painting (3.78 m by 4.53 m) at high resolution over such a long period of time included the ability to plan and collect a high number of swaths, to maintain a stable illumination system during the data collection and to keep the hyperspectral cameras in focus, given that the painting deviates by 3 cm from a plane. Thus, a 3-D computer-controlled imaging frame (Figure 1), that could hold each hyperspectral camera (as well as other imaging devices) along with the illumination system and the collection computer, was designed and constructed. Given the size of the imaging frame, a separate computer was needed on the floor for the scientists to control the dedicated computer belonging to each of the hyperspectral cameras. Further, a sophisticated position-based control system was needed to make sure the image swaths were collected with sufficient overlap to allow the final 3-D image dataset to be mosaicked together. To keep the cameras in focus and without change in the spatial sampling (i.e., optical magnification) during the scans, a third axis of motion normal to the painting was needed along with an automatic feedback system. Finally, to minimize the effect of the temporal drift of the dark response of the hyperspectral cameras and the intensity of the illumination system, frequent dark frames and measurements of the diffuse white reflectance standard were needed. The VNIR 3-D image data collection had to be done at night, since the painting remained on view to the public during the day and the room lights had to be turned off to avoid interference. Taking all this into consideration, to cover the entire surface of the painting, even with an imaging frame that meets the above requirements, collecting a full RIS image cube would take at least two months.

Another challenge is that, once the RIS swaths, both VNIR and SWIR, were collected, they needed to be mosaicked and registered to a reference color image to create two apparent reflectance image cubes of the painting, one spanning the VNIR and another spanning the SWIR. The registration step of the two RIS cubes to a color image was key to ensuring that the spatial features in the RIS data could be directly compared with those in the MAXRF data cube and other datasets. To do this, a novel software algorithm to perform the mosaicking and registration had to be developed, coded and tested.

Once the two final RIS image cubes (VNIR and SWIR) were mosaicked and registered, the 3-D image cube was analyzed by examining false color images and using multivariant spectral imaging processing algorithms to classify the image cube into a series of reflectance spectral endmembers (characteristic spectra). The reflectance spectral endmembers were then used to produce maps of spatial regions where the spectra match those of the endmembers. The analysis of the spectral features of the reflectance endmembers along with other analytical data allowed for the determination of which pigments made up the spectral endmembers. Thus, in the end, the spectral endmember maps could be interpreted as maps of pigments. The SWIR portion of the RIS cube was also investigated for evidence of preparatory sketches or changes in the painted composition. This information, when combined with results of other imaging methodologies, will provide new insights into how Rembrandt created this masterpiece and how it can be best conserved.

In this paper, the technical aspects of the RIS collection plan, imaging frame hardware and hyperspectral cameras used are reported together with the results obtained from the figure of lieutenant Van Ruytenburch as an example of the achievable information and the quality of the full RIS 3-D image data obtained on *The Night Watch*.

## 2. Materials and Methods

### 2.1. Painting and 3-D Imaging Frame

*The Night Watch* painting was mounted vertically on a custom easel which is attached to a load-bearing wall. In front of the painting, a custom 3-D computer-controlled mechanical scanner (or imaging frame) was mounted to hold and move the different scientific instruments across the surface of the painting (Figure 1). To minimize the impact of vibrations, the imaging frame was fixed to the wall on which the painting was mounted. This was necessary to minimize vibrational differences since some of the imaging modalities have spatial resolution of a few micrometers. This entire setup is enclosed in a glass chamber (Figure 1) to provide visitors to the museum with a glimpse into the ongoing scientific analysis as well as the painting itself.

**Figure 2:**
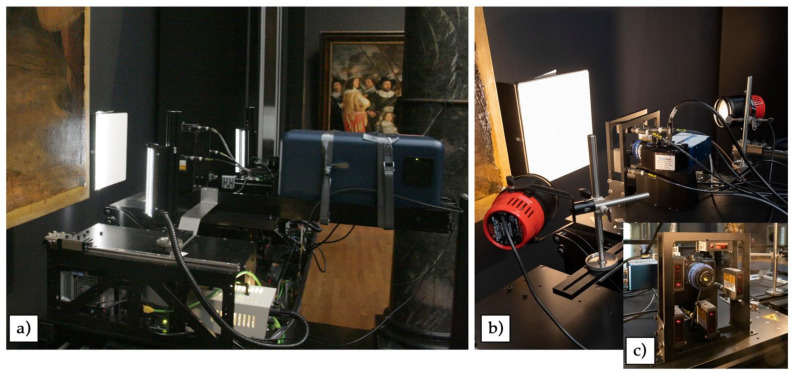
The line scanning reflectance imaging spectrometers used to scan *The Night Watch*: the (**a**) VNIR camera (400 to 1000 nm) and illumination system and the (**b**) SWIR camera (900 to 2500 nm) and illumination system, anchored to the instrument platform in front of the white spectralon target. The servo system with five range finding lasers (top, bottom, left, right, lower right corner) used to control the focus is visible in the zoom-in (**c**).

The imaging frame (Figure 1) consists of a custom vertically mounted 2-D (x–y) gantry scanner (Segula, now TBRM Engineering Solutions, NL) with a scanning area of 5.4 m × 4.4 m to allow for the full surface of the painting to be accessed. The instrument platform on the x-beam (Figure 1–red-dot line) of the imaging frame allows for the mounting and moving of line scanning systems, such as hyperspectral cameras, and step/stare framing cameras. The instrument platform also holds the computers which run the instruments, although they are controlled remotely from the ground. Since the painting surface deviates from the nominal *x*–*y* imaging frame plane by up to 3 cm, the instrument platform allows for a third axis motion (*z*-axis, toward and away from the painting) as well as pitch(rx) and yaw(ry) motions (these 2 rotational movements are with respect to a virtual pivot point on the painting, being the focus point of the device). Thus the x–y motion can place a camera at any point on the painting, the z axis can set focus and the yaw and pitch motion of the platform can ensure that the optical axis of a camera is normal to the painting surface. To keep the cameras aligned and in focus (fixed distance in z from the painting), a closed loop servo system with five range-finding lasers (top, bottom, left, right, lower right corner), with centered wavelength at 655 nm and an image-based laser sensor with 15 measurement points spread over the image area (Keyence), is used (Figure 2c).

The imaging frame supports several data collection modes, including step/stare for imaging with framing cameras and constant speed scan mode for line scanning cameras. If the latter set-up is used, trigger pulses, based on time, are sent to the hyperspectral camera to collect the image lines. The imaging frame has encoders that provide accurate and repeatable spatial positioning in x,y,z (±0.01 mm). The data collection in any of the two collection modes (step/stare or constant speed scanning) is fully programmable, and the operation of the imaging frame is preformed using a GUI. This allows the user to position the platform at any point on the painting and initiate one of the collection modes, including whether the auto focus and auto alignment of the camera to the local painting surface normal is turned on or off. The GUI also allows for the automatic collection of reference target data, such as calibration targets.

Finally, the imaging frame is equipped with a unique and proprietary full range of safety mechanisms and features, including object detection/collision avoidance.

### 2.2. Reflectance Imaging Spectrometers

A reflectance imaging spectroscopy from 400 to 2500 nm was performed using two line scanning hyperspectral cameras: one that covers from the visible to the near infrared spectral range (400–1000 nm, VNIR in Figure 2a) and the other from the near infrared through the short wavelength infrared (900 to 2500 nm, SWIR in Figure 2b).

The VNIR hyperspectral camera (Surface Optics Corp., San Diego, CA, US—710E model) utilizes a grism imaging spectrometer combined with a backside illuminated EMCCD (Princeton Instruments) which provides 1024 spatial pixels along the slit. It is equipped with a LM50HC-IR lens by KOWA with an EFL of 50 mm. The spectral range of the camera is 400 to 1000 nm and a spectral resolution of 2.54 nm, with a total of 256 spectral channels, and operates at an F-number of 2.8. With the working distance of 70 cm, the spatial resolution at the painting was 0.168 mm, resulting in a vertical field of view of 172.03 mm and a depth of focus of ±2 mm. The integration time set on the camera was 200 ms per spatial line, and the scan speed used was 0.672 mm/s (Table 1). The illumination is provided by two 20 cm line lights with cylindrical lenses (Schott fiber optic line light) set up at 50 degrees from the painting normal and at a distance of 40 cm from the painting surface (Figure 2a). The fiber bundle of each line light was illuminated using Euromex EK-1 light sources containing Solux 4700 K, 50 W THL bulbs which are coated to minimize the emission of UV and IR light. These two-line lights produce a 20 × 2.5 cm vertical rectangle patch of light on the painting, whose brightness is 1300 lux. The use of these lights assures a lower net light exposure of the painted surface and at the same time a reduced amount of specular reflection from the painting varnish. A “dark” image cube (meaning a data cube where no light is allowed to reach the detector array) is automatically collected by the system, while a “white” image cube consists of a swath with 1024 lines collected from the diffuse white reflectance standard (99% reflector, Labsphere, Inc., North Sutton, NH, US) that was mounted in the plane of the painting, fixing it to the easel holding the painting to the wall. The subtraction of the dark from each acquisition (painting or white reference) is provided by the SOC software, while the apparent reflectance image cubes are calculated, through a process called flat fielding, by dividing each swath collected by the “white” image cube of the reflectance standard after the dark cube subtraction.

The SWIR hyperspectral camera uses a reflection grating imaging spectrometer coupled with an MCT detector array (M640, Headwall Photonics, Boston, MA, USA) with 640 spatial pixels along the slit. The fore optic lens has an f-number of 2.5 and an EFL of 25 mm. The spectral range is 900 to 2500 nm, with a spectral resolution of 6 nm and a total of 267 spectral channels. The spatial resolution at the painting was 0.168 mm, and the vertical field of view at the painting was 107.52 mm. The depth of focus at the painting was ±2.4 mm. The integration time set on the camera was 110 ms, and the scan rate used was 1.12 mm/s (Table 1). The illumination of the painting was achieved using an Ianiro Gulliver30 lighting kit with 150 W halogen 3000 K bulbs (Osram), also set at 50 degrees from the surface normal at a distance of 25 cm (Figure 2b). The light level was controlled using a rheostat dimmer and set to provide 12 to 14 W/m^2^ at the painting surface (light meter with thermal radiator sensor by Preservation equipment—684–775 C). The calculation of the apparent reflectance was provided directly by the Headwall acquisition software, using the most recent dark and white reference cubes to minimize the drift of the MCT array.

### 2.3. Construction of the Two Reflectance Image Cubes and Spectral Data Analysis/Treatment

The reflectance image swaths were spatially registered individually to a reference color image of the painting (20 µm sampling resolution [13]) and blended into a single whole-painting spectral data cube. The algorithm proceeds broadly in the following steps. First, a pair of convolutional neural networks (CNNs) is trained to learn a pair of nonlinear image filters, one of which is applied to the data cube of the RIS swath and the other of which is applied to the high-resolution visible image (color). The training objective is based on a contrastive loss computed on the normalized spatial cross-correlation of the filtered images, so that the CNNs learn filters in such a way that there is only one relative offset of the locally filtered data cube that aligns well with the filtered visible image, as measured by the difference between the maximum normalized cross-correlation and the mean normalized cross-correlation between the filtered image pairs. In other words, the performance of the filter pair is measured by the degree to which the normalized cross-correlation is strongly peaked at a single location, with all other relative offsets having a poor relative correlation between the two, thereby addressing the problem that may arise when there are many relative offsets of comparable quality. Thus, the networks learn how to extract features that are shared by both imaging modalities (e.g., cracks or visible portions of brushstrokes) while suppressing features present in only one. Starting with an approximate manual positioning of the RIS swaths on the corresponding location of the whole-painting visible image, the CNNs are applied to a decomposition of the overlapping portions of the images into dense (overlapping) square image tiles, thereby enabling us to compute the relative x- and y-offsets from each RIS tile to the corresponding visible image tile. This results in a deformation map across the entire RIS swath that indicates how it should be locally displaced to align with the visible image. The overall deformation is decomposed into homographic plus residual terms, which are then recorded and applied to the RIS swath to improve its alignment to the visible image. The procedure is repeated iteratively to refine the RIS swath placement until the maximum necessary displacement is less than 1 pixel (3 iterations are typically needed). The sequence of derived transformations from the refinement iterations are mathematically combined into a single overall geometric transformation and applied in a single pass to all spectral channels of the RIS swath using a high-order interpolation kernel. This procedure is repeated for each of the RIS swaths, resulting in a collection of separate deformed swath images. In the next step, the final geometries of the overlapping swaths are analyzed to assign “ownership” of each pixel in the overlapping areas to the individual swath whose “soft center” (as computed by solving a constant-source Poisson equation) is nearest to the given pixel. Finally, the individual swaths are blended together according to the ownership mask using a Gaussian-Laplacian multiresolution blending algorithm, that blends large-scale trends in the swaths together over a long-length scale and small details together over a short-length scale. This ensures smooth transitions from one swath to another in overlapping regions where the swaths disagree on the relative brightness of a given pixel while also retaining a sharp detail. The end result is a single whole-painting RIS data cube that is precisely registered to the high-resolution visible image. A similar procedure is followed for the other imaging modalities (radiography, MAXRF, UV-induced visible fluorescence, etc.), so that all of the images are mutually co-registered.

For the VNIR reflectance image cube analysis of the selected detail of the figure of lieutenant Van Ruytenburch, a first derivative reflectance image cube was calculated as the transition edges and absorption features became more clear in the spectra [14]. To do this, the VNIR detail cube was first spatially averaged (2×) in order to reduce the random noise and improve the signal to noise ratio. The derivative image cubes were constructed using a Matlab script (MathWorks ^®^Natick, MA, USA) with a window of 3 spectral pixels. To find the spectral endmembers, or characteristic spectra, a section of the coat of the figure of lieutenant Van Ruytenburch was analyzed. The glints from the distance measuring lasers (emission at 655 nm), which are present at the edges of each swath, were found using a spectral anomaly detection algorithm ENVI (L3 Harris Geospatial). Once the spatial pixels having the sensor laser glints were masked out, the endmembers were mainly found through the use of Spectra Hourglass Wizard (ENVI-SHW), although a few were also found manually. These formed the set of spectral endmembers used to make maps for the figure or for the details. In each case, the maps were made using spectral angle mapper (SAM) in ENVI. The maps were obtained by restricting the spectral range used for each endmember to span the region that had the most characteristic features of the material of interest. The SAM angle tolerance used was selected manually from the histogram of the ENVI SAM Rule image by checking to make sure the highlighted pixels contained the spectral feature of interest. This approach favors the spatial pixels having the highest amount of a given pigment and, as such, is often a conservative estimate of where the pigment is present.

For the SWIR data cube, eliminating the laser signals was not necessary, as the laser emission is outside the spectral range of the camera. The random noise was reduced through a principal component analysis (PCA) of which PCA bands 1 to 8 were kept and the inverse PCA was applied to obtain the filtered SWIR data cube. From this data cube, the false color image was constructed. To map the hydroxyl features in the 1300 to 1600 nm region, the continuum removal algorithm in ENVI was used. To reduce the presence of “hot pixels”, the Distripe algorithm in ENVI was applied. The endmembers were found using the ENVI-SHW, and SAM maps were made using the full spectral ranges from 1300 to 1600 nm and 2200 to 2400 nm. The SAM tolerance angle was set using the method described above.

## 3. Results and Discussion

### 3.1. The Collecton of the RIS Swaths and Hyperspectral Camera Perfromance

Due to the many challenges previously addressed, among which the large dimensions of the painting and the relatively small field of view of the VNIR and SWIR line scanning hyperspectral cameras (172.03 mm and 107.52 mm), the data collection was performed over the course of two months. The data collection with each hyperspectral camera was performed as a number of image swaths organized in rows and columns. The height of each swath is determined by the height of the slit of each camera projected onto the painting. The maximum of lines in a single swath, which spanned 4.53 m, the full width of the painting, is 26,964 lines, which would exceed the RAM storage capacity of the computer running the cameras, and thus the length of a given swath was determined by the available memory. The total number of rows required depended not only on the height of the painting, and the swath height on the painting, but also on the amount of overlap needed to ensure no gaps: a 10% overlap for VNIR and a 14% overlap for SWIR. The full collection parameters for the hyperspectral cameras are given in Table 1. The vertical overlap, with the left and right swath, was kept at 2% for both VNIR and SWIR.

For the VNIR scanning, the optimal number of swaths across the painting (or columns) was 3, and the number of vertical rows was 26. One row of VNIR data consisted of 30 GB of data, giving a total 780 GB of data collected for the entire painting. VNIR swath acquisitions were performed over the course of 12 nights, since the room lights needed to be off to avoid interference in the spectra. To determine the amount of variation in the illuminating light from the line lights during the 12 days (or sessions of measurements), the image cubes collected onto the “white” standard panel were compared. These results showed that the percent value of the standard deviation was lowest in the 500 to 990 nm spectral range (2 to 3%) and highest at the edges of the spectral range (5% at 400 and 10% at 1000 nm). This small variation in the absolute light level was not an issue, given that the detector response is linear over this range, since the exposure was set to ~80% of the full well. The impact of these small variation was adjusted in the registration/mosaicking image processing step. The noise in terms of units of reflectance from the white standard was calculated for the VNIR spectra. The root means square (rms) noise in reflectance was found to vary from 0.01% at the edges of the spectral range to 0.006% in the center of the spectral range.

For the SWIR scanning, the optimal number of swaths or columns was also 3, and the number of rows collected was 41, since the field of view is smaller than the VNIR hyperspectral camera. The SWIR swaths could be acquired during daytime since the overhead lights in the gallery had little SWIR radiation. The total number of days to collect the SWIR data was 19. One row of SWIR data is 19 GB, giving a total of 779 GB of data. The stability of the illumination system over the total collection time of 19 days was analyzed in the same way as the VNIR, and the variation over the spectral range was 1.4 to 2%. The value of 2% occurred around 900 nm. The spectral noise in terms of rms about the diffuse white reflector was found to vary from 0.01% (in particular, 0.018 at 2500 nm) at the edges of the SWIR spectral range to 0.006% in the center of the spectral range.

### 3.2. Automated Focus System during the RIS Line Scanning

A previously measured height map of *The Night Watch* shows that the painting deviates from the nominal x–y plane of the imaging frame by as much as 3 cm (upper left corner). This is due to deformations in the canvas associated with the wooden stretcher and the painting not being mounted parallel to the wall. Although, due to Rembrandt’s frequent use of impasto, the paint thickness can vary locally up to a few mm, this is within the depth of focus of the hyperspectral cameras. The deformation of the canvas associated with the stretcher and the mounting to the wall varies slowly across the painting relatively to the height and length of the swath of the hyperspectral cameras. Thus, the autofocus system is used to keep the cameras “in focus” during the scans to account for these distortions. Instead of refocusing the fore optic lens of the hyperspectral cameras, which would result in a magnification change, the hyperspectral cameras were moved toward or away from the painting (*z*-axis). The servo system, described above, utilized the distance information from sensor lasers positioned on a specific frame anchored to the moving platform, between the spectrometer and the surface of the painting (Figure 2). The sensors’ frame for the VNIR and the SWIR spectrometers was adapted to the two different fields of view. For the VNIR camera, one laser sensor (bottom) was used to minimize the interference of the laser in the spectral data, while for the SWIR two sensors were used (top and bottom). The rate of correction was 0.2 Hz, which, at the scan speeds (Table 1), along with the distance measurement accuracy, ±0.01 mm, ensured that the cameras remained in good focus. This can be seen by the fine paint cracks observed in each of the scans. The laser emission signal in the spectra was masked in the data cubes as explained above in the data cube processing section.

### 3.3. Analysis of the RIS VNIR and SWIR Image Cubes and Spectral Image Products

After the collecting the 78 VNIR and 123 SWIR swaths and converting the data in apparent reflectance, a novel image registration algorithm, described in the Materials and Methods’ paragraph 2.3, was used to automatically register and mosaic the VNIR and SWIR image swaths onto a previously generated 20-µm resolution color image of the painting. The spatial accuracy between the RIS images and the color image is sub-pixel. The resulting VNIR reflectance image cube measures 28,907 by 24,219 spatial pixels by 260 spectral bands (780 GB), and the SWIR reflectance image also measures 28,907 by 24,219 spatial pixels and 267 spectral bands (765 GB) (Table 1).

**Figure 3:**
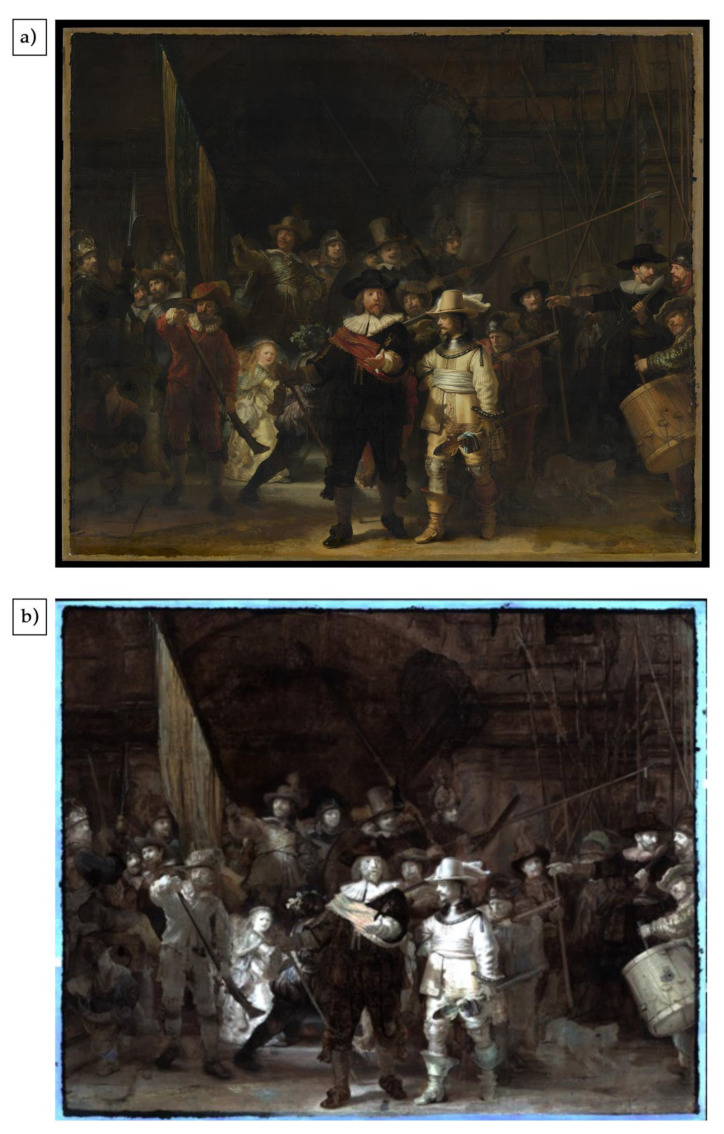
Accurate color image calculated from the VNIR reflectance imaging dataset using D65 standard illumination (**a**) and a false color image of the SWIR image cube prepared using 1603 nm, 1201 nm and 1040 nm spectral bands for the RGB channels (**b**).

An accurate color image has been calculated from the VNIR reflectance image cube using D65 standard illumination (Figure 3a) and the standard observer functions. To reduce the small variations in brightness associated with each of the swaths, the blending algorithm combines them using a multiresolution spline. A false color of the SWIR image cube was prepared using 1603 nm, 1201 nm and 1040 nm spectral bands for the RGB channels (Figure 3b).

**Figure 4:**
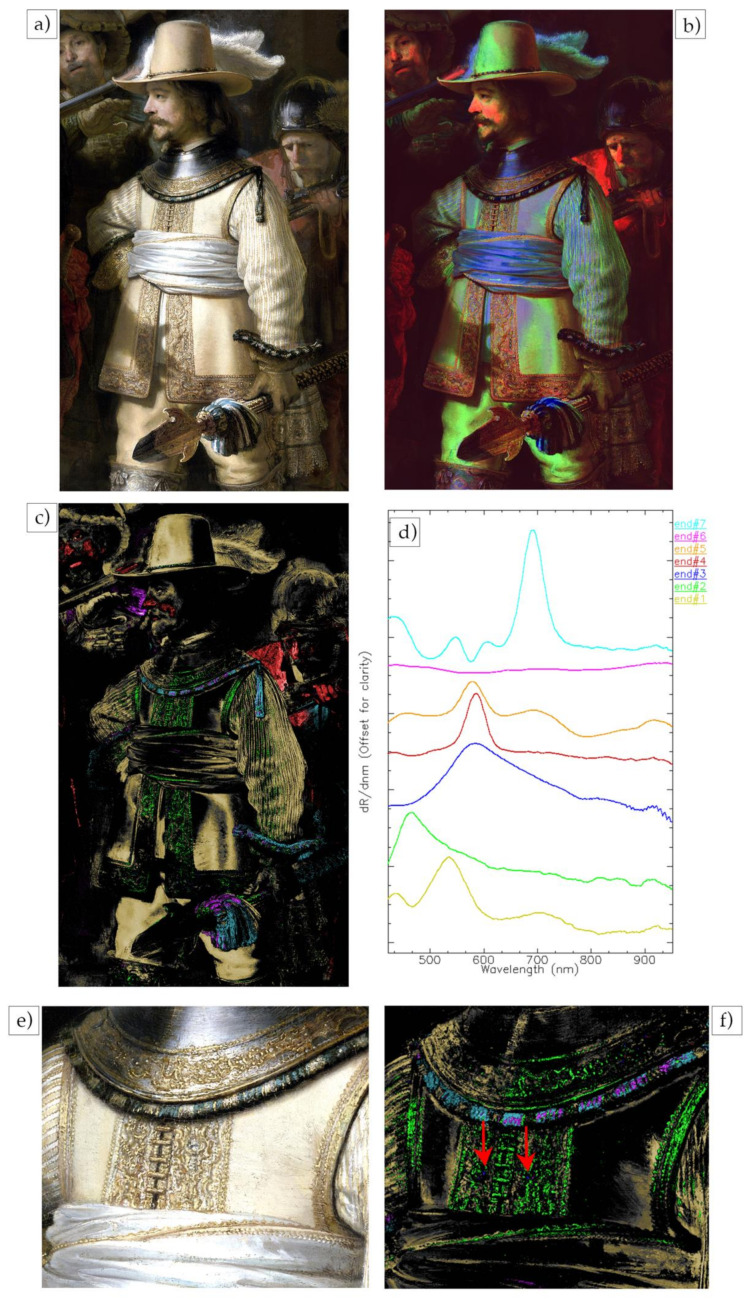
Color image of the figure of lieutenant Van Ruytenburch (**a**), 1st derivative false color of the VNIR cube with 620 nm, 580 nm and 520 nm bands used for the RGB channels (**b**), SAM maps (**c**) for the seven reflectance endmembers (**d**). Color image of the detail of the embroidery of the coat of lieutenant Van Ruytenburch (**e**) and SAM map (**f**) of the same detail.

The preliminary analysis of reflectance image cubes of the painting has focused on two aspects. First, the identification and mapping of pigments for the main figures, and second, the preparatory sketches and compositional changes revealed by the false color SWIR image of the entire painting. In this paper, the analysis of the reflectance data of the figure of lieutenant Van Ruytenburch (Figure 4a) is presented to demonstrate the quality of the image cubes and the ability to identify pigments through their spectral features and map their distribution.

Rembrandt’s paintings are challenging for reflectance imaging spectroscopy, given their overall darkness and consequent low reflectance (<15%). Secondly, the intimate mixing of the pigments in the paints masks the spectral features associated with each individual pigments, as does the contribution of the light scattering from the pigments themselves. Thus, the 1st derivative of the VNIR reflectance image cube was used to emphasize the spectral features, to improve the identification and ultimately map the specific pigments. This advantage can already be seen in the false color made from VNIR’s first derivative image cube using 620 nm, 580 nm and 520 nm bands for the RGB channels (Figure 4b). The false color derivative image visually enhances the separation of pigments in comparison with the visible image. The false color immediately shows a clear difference between the color of the dark areas of the coat, which are bright green, the white sash, that has a blue tone, and some areas of the face, that are bright red. The inspection of the first derivative reflectance spectra shows that green is associated with a yellow ochre, blue with lead white and red with vermilion, although false color maps are not the best method to get an accurate distribution of the pigments.

A multivariant statistical algorithm (ENVI-SHW), described in the Materials and Methods section, was used to classify the image cube into a set of endmember spectra, which were used to make maps. From the analysis, a total of seven endmembers (Figure 4d) were found. These spectra are dominated by one pigment mixed with some white and mixture of pigments are not considered. These endmembers were used for all the pigment maps made of the whole figure and the details. The pigments found are consistent with those in Rembrandt’s palette, and many had been independently identified using other analytical methods. The first endmember (end#1 in Figure 4d) has the spectral characteristic features of goethite (α-Fe^III^O(OH)), the iron-based chromophore present in yellow ochre, having a maximum peak in the derivative spectrum at 535 nm, together with other, more broad peaks at 440, ~700 and 920 nm [15]. This endmember maps to the darker yellow hues of the buff coat, the hat and the lightest areas of the flesh (yellow in the SAM map, Figure 4c). The second endmember #2 (end#2 in Figure 4d) has the spectral features associated with a lead-based yellow semiconductor, lead-tin yellow (Pb_2_SnO_4_ type 1, Pb(Sn,Si)O_3_ type II), with a maximum peak at 460 nm [16]. It maps the highlights of the buff coat (green in the SAM maps in Figure 4c,f) that are correspondent to the light-yellow impasto areas of the entire embroidery besides the shadow, which is created by the hand of the figure standing on the left. The position of the peak in the derivative spectra suggests that the pigment is lead-tin yellow I (reflectance max around 480 nm) rather than II (reflectance max around 500 nm) [16], although the MAXRPD analysis gives a more definitive assignment (data not shown). Endmember #3 (end#3 in Figure 4d) maps to the small orange dots in the embroidery (blue in the SAM map in Figure 4f indicated by red arrows) and its spectrum shows an asymmetric peak at 570 nm and is consistent with realgar (As_4_S_4_) [16], although a definitive assignment is only possible when other information is combined. Specifically, in these areas, arsenic (As) has been detected through MAXRF measurements (data not shown). The endmember 4 (end#4 in Figure 4d) has a nearly Gaussian shape with a symmetrical narrow peak at 587 nm and a FWHM of ~40 nm characteristic of a semiconductor pigment [17]. Since the peak, or inflection, is at 587 nm, this can be assigned to vermilion (HgS) rather than red lead [18]. This endmember maps to the reddish hues of the flesh tones of the figure of lieutenant Van Ruytenburch and of the musketeer behind him (red in the SAM map in Figure 4c). The remaining endmembers are of blue pigments. Endmember #7 (end#7 in Figure 4d) has absorption features characteristic of smalt, a potassium glass containing cobalt. It can be identified from the three maxima in the first derivative spectra at 549, 604 and 691 nm [19]. This endmember maps to the collar, sleeve and spear decoration (cyan in the SAM map in Figure 4c,f). A second blue pigment, which is represented by endmember #6 (end#6 in Figure 4d), has the typical slow rise in reflectance after 640 nm [16] of azurite, a copper-based carbonate [Cu_3_(CO_3_)_2_(OH)_2_]. The confirmation of the assignment to azurite can be made from specific absorption features observed in the SWIR spectral region (see below). The endmember spectra maps to some portions of the blue collar and the spear decoration and, interestingly, to the left area of the face of lieutenant Van Ruytenburch (puprle in the SAM map in Figure 4c,f), corresponding to the green-blue costume of the character standing behind him.

**Figure 5:**
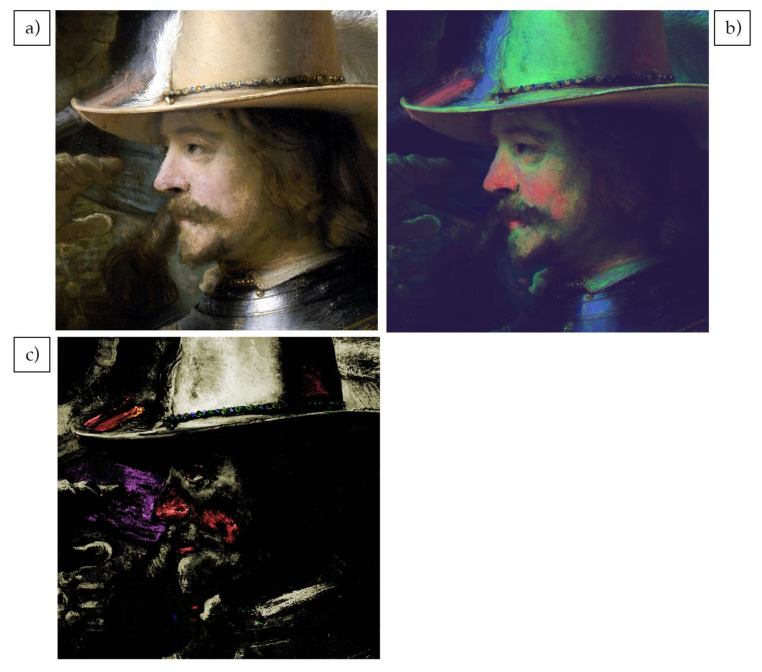
Color image of a detail of the face of lieutenant Van Ruytenburch (**a**), 1st derivative false color of the VNIR cube with 620 nm, 580 nm and 520 nm bands used for the RGB channels (**b**), SAM maps (**c**) for endmember #1 in yellow, #4 in red, #6 in pink and #5 in orange.

The high spatial resolution and quality of the spectral data can be seen in the maps of the details of the embroidery (Figure 4e) and the head (Figure 5a) of lieutenant Van Ruytenburch. In the SAM pigments map of the detail of the coat (Figure 4f), it is possible to observe how precisely the embroidery is mapped: the green map (lead-tin yellow) corresponds to the small dots of light yellow impasto paint used in the light areas of the embroidery. It contrasts with the shadow areas of the collar, where these light yellow dots are almost entirely absent. Individual orange small dots can be seen in the detail of the embroidery (Figure 4e) and were mapped as realgar (blue spots in Figure 4f indicated by red arrows). The blue decoration of the collar is also mapped in detail, showing the presence of bright blue brushstrokes containing azurite (purple) adjacent to dark blue shadow areas containing smalt (light blue).

Looking at the SAM map of the detail of the head (Figure 5c), it is clear that Rembrandt used yellow ochre mainly in the areas of the flesh tones exposed to light, while the areas in the shadow of the head have a different composition. To achieve a more blushed color, vermilion was used on the nose and cheek. Yellow ochre is used for flesh tones and is also present in the white feather on the right of the hat, most likely to achieve a warmer tone. Due to the spatial resolution, it is also possible to understand the composition of the hat garland, that was realized applying tiny brush strokes of lead-tin yellow, smalt and ochre (Figure 5c).

Adjacent to the head of Van Ruytenburch is the fire coming out of the gun shot by one of the musketeers. The map shows that the flame is mostly achieved with vermilion, but Rembrandt has added some red ochre as well (Figure 5c), which was identified from endmember #5 (end#5 in Figure 4d). This endmember has a maximum peak in the derivative spectrum at 580 nm and another, more broad peak at ~700 nm, typical of red ochre (hematite chromophore α-Fe_2_O_3_) [15].

**Figure 6:**
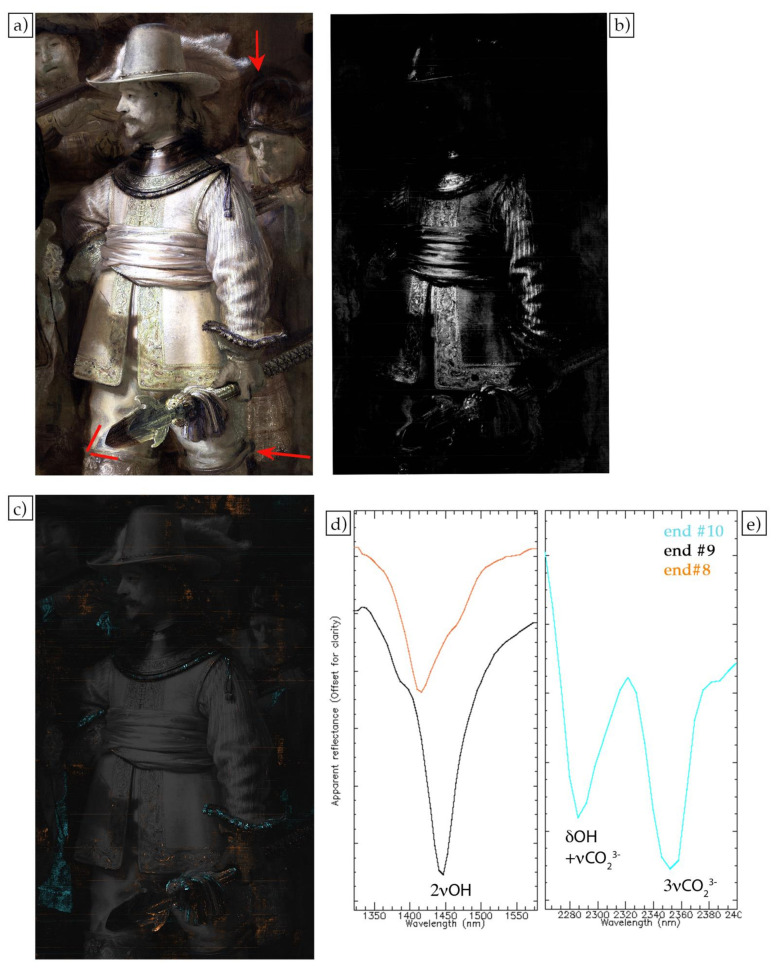
SWIR false color (1603 nm, 1201 nm and 1040 nm) of the figure of lieutenant Van Ruytenburch (**a**), SAM map (**b**) for endmember #9 (**d**) and SAM maps (**c**) for endmembers #8 and #10 (**d**,**e**), showed here on top of the darkened false color.

In the SWIR false color image (1603, 1201, 1040 nm), many of the pigments become more transparent, i.e., less absorbing, and the effect of the light scattering from particles dominates, including the scattering from the ground layer as well as the absorption from infrared-absorbing pigments such as carbon-based blacks (Figure 6a). This allows for the visualization of possible preparatory sketches (containing carbon-based black) and changes in the painted composition. An example of the latter can be seen in the figure analyzed, namely a change in the position/length of the tip of the spear held by Van Ruytenburch (red lines in Figure 6a). In this case, the pigments used on top of the first design become transparent in the infrared region, revealing the old position or length of the spear tip. Dark or black painted sketch lines can be observed in Van Ruytenburch’s proper left leg (see the red arrow in Figure 6a), where the pants meet the boots, and also in the helmet of the figure on his right (see the red arrow in Figure 6a).

The SWIR spectral range is particularly useful to identify functional groups in molecules that present vibrational modes in this region [20], for example hydroxyl, carbonates and C-H groups. In the spectral region between 1300 and 1600 nm, the hydroxyl (OH) stretching overtone can be observed. The graph in Figure 6d shows two different endmembers presenting a second overtone of a stretching vibrational mode (2νOH), with absorptions at different wavelengths that allow for them to be assigned to specific pigments. Because these vibrational absorption features are narrow (~50 nm wide), a continuum removal method, as specified in the experimental section, was applied to the 1300 to 1600 nm spectral region of the cube to better isolate them from the broad spectral variations from light scattering and absorption. Endmember #9 (end#9 in Figure 6d) has an OH overtone at 1450 nm, which is assigned to hydrocerussite [Pb_3_(CO_3_)_2_(OH)_2_] [10], one of the two main constitutive phases of lead white. This endmember maps to the brightest white areas of the coat, the sash and the feather on the left side of the hat (white areas in the SAM in Figure 6b). It is either the dominant pigment, such as in the white feather and in the sash, or mixed with another pigment, such as with the yellow ochre of the coat (while in the previous discussion of the VNIR spectral range lead white was not uniquely identified, the presence of a white pigment was clearly seen in the reflectance spectra showing a strong increase in reflectance starting from 400 nm).

Endmember #8 (end#8 in Figure 6d) shows an absorbance feature at about 1415 nm, which is in the region for the OH groups contained in clay minerals. The position of the OH overtone at 1415 nm and the absence of the characteristic triplets (1395, 1405 and 1415 nm) [21] confirms that the clay does not belong to the class of kaolin, and the best correlation is with a clay of the class of mica, specifically muscovite (Al_2_K_2_O_6_Si) [22]. This endmember maps to the medium brown-colored areas in the SAM map in Figure 6c. Clay minerals are commonly found in earth pigments, such as ochre, sienna or umber [23]. Earth pigments can be used as pigments in the paint layer or in a colored ground. A prior analysis showed that the ground of *The Night Watch* is a quartz ground, which also contains small quantities of earth pigments in addition to quartz and clay minerals [24]. In the area above Van Ruytenburch, the medium brown-colored areas seem to be associated with the ground layer (they also show the canvas structure) (Figure 6c). At the same time, in other areas, specifically on the tip of the spear, there is an accumulation of this clay, suggesting the use of earth pigments in the paint layer.

The SWIR dataset was also explored in the region between 2200 and 2400 nm, where specific overtones and combination bands of functional groups can also be observed. Endmember #10 (end#10 in Figure 6e) presents two spectral features at 2285 and 2352 nm, respectively associated with the combination band of carbonate stretching and hydroxyl bending (δOH + νCO_3_^2−^) and the carbonate stretching’s third overtone (3νCO_3_^2−^) of azurite [Cu_3_(CO_3_)_2_(OH)_2_] [25]. This endmember maps to the decoration of the collar, the sleeve and the spear and, furthermore, to the costume of the character in the back (cyan areas in the SAM map in Figure 6c). This result confirms the azurite map obtained from the VNIR region (Figure 4c). Still, the azurite map obtained in the SWIR region shows that more azurite is present than indicated by the VNIR map, for example in the areas related to the characters in the back. The VNIR spectral region is more sensitive to colors (due to electronic transitions) and gives information about materials present on the surface, while the SWIR has a higher depth of penetration into the paint layers and the spectral features are less related to the colors. There are several possibilities: the areas where we see the azurite only in the SWIR could be related to a layer of azurite underneath, not visible with the VNIR, the azurite could be mixed with a dark pigment, making it hard to see the electronic transition in the visible region, or the azurite could be darkened due to degradation processes (darkening of the binding medium, formation of oxalates, etc.). In this case, it seems that the region is dark because it corresponds to the shadow of the figure on the left. In addition, in the SWIR false color image, this area appears darker than other azurite-containing areas, indicating the possible presence of an infrared-absorbing pigment such as carbon black or bone black, used to create the shadow. In summary the SWIR image cube not only provided information about the presence of preparatory sketches and changes in the painted composition but also allowed for a more extensive mapping of some pigments than was possible in the VNIR dataset—specifically, those pigments which have vibrational features in the SWIR (azurite and lead white)—and, perhaps most importantly, or the identification of a clay mineral contained in the ground.

## 4. Conclusions

The aim of this paper was to describe the planning, technical aspects and results of the reflectance imaging spectroscopy (RIS from 400 to 2500 nm) campaign performed on a large-scale painting, namely *The Night Watch* (3.78 m by 4.53 m). The RIS line scanning of the entire painting was accomplished using two different line scanning spectrometers (VNIR from 400 to 100 nm and SWIR from 1000 to 2500 nm) sequentially. Despite the canvas’ deviation from a plane, the RIS final image cube is sharp and the spatial resolution appears constant at 168 μm. This was achieved thanks to the use of a novel 3-D-computer controlled imaging frame that allowed not only for the movement in y and x of a platform to which the cameras were mounted, but also for the automatic control of the focal distance of the cameras (z movement), maintaining a constant focus during the line scanning acquisition. Despite the challenges of scanning throughout multiple days, keeping the light level constant and the cameras in focus, and operating the cameras remotely from a workstation on the ground, almost 2 TB of data have been acquired and stored on the Rijksmuseum server. The swaths have been stitched and registered to a visible image using a novel algorithm. VNIR and SWIR reflectance spectral data show a good signal to noise ratio, allowing for the calculation of the first derivative. The endmembers were associated to different pigments and used to map those pigments on the surface of the painting.

The VNIR RIS dataset was useful to identify the pigments present in *The Night Watch*, such as vermillion, lead tin yellow, azurite, realgar, smalt, goethite and hematite, which are consistent with Rembrandt’s palette. The high spatial resolution of the scanning allowed for the discrimination of the pigments used for small brush strokes in some details, for example the hat and the embroidery of lieutenant Van Ruytenburch. The SWIR false color allowed for the clear visualization of changes in the painted composition. The SWIR line scanning sensor helped identify and map separate pigments based on the vibrational features. This was especially important in finding azurite in dark passages, in which it could not be seen in the VNIR data but was easily mapped by the vibrational features in the SWIR. This observation may even indicate areas of degradation, which will be the object of future studies. The ability to non-invasively identifying and mapping a clay mineral in the ground, as well as identifying areas where the ground is relatively exposed, is a new finding that SWIR imaging offers. On the other hand, reflectance imaging spectroscopy in the SWIR can only map the hydrocerussite phase of lead white, through the OH overtone feature, and thus cannot contribute to the understanding of whether the two phases of lead white (cerussite and hydrocerussite) were selectively used in the composition.

We have demonstrated a successful experimental protocol to create high spatial resolution RIS image cubes, that span the 400 to 2500 nm spectral region, of a very large painting. Moreover, because the final cubes are registered to a reference color image, the other imaging modalities can be directly compared to the RIS images to lead to new insights into Rembrandt’s painting technique, the materials used and the state of conservation of *The Night Watch*. Such information is of great importance to help guide a conservation treatment for this important masterpiece.

## Figures and Tables

**Table 1:** Parameters used during the VNIR and SWIR-RIS scanning of *The Night Watch*.

	VNIR	SWIR
Distance from camera to painting	680 mm	280 mm
Vertical field of view set by slit	172.032 mm	107.52 mm
Spatial resolution at the painting	0.168 mm	0.168 mm
Scan speed	0.672 mm/s	1.12 mm/s
Overlap between rows (v), columns (h)	v: 90 lines–10% h: 238 lines–2%	v: 92 lines–14% h: 119 lines–2%
Number of rows	26	41
Number of columns	3	3
Total number of swaths acquired	78	123
Days	12	19
Dataset dimension after flatfielding	780 GB	779 GB
